# A Case Report of Possible Health Benefits of Extra Virgin Olive Oil

**DOI:** 10.1100/tsw.2003.112

**Published:** 2003-12-05

**Authors:** Said Shahtahmasebi, Shahnaz Shahtahmasebi

**Affiliations:** School of Mathematics and Statistics, Faculty of Health and Sciences, Christchurch Polytechnic Institute of Technology, POBox 540, Christchurch, New Zealand

**Keywords:** diet, nutrition, olive oil, coronary heart disease, cholesterol, public health

## Abstract

The literature on the chemical analysis of cooking oils suggests that the cholesterol-reducing effect may well be due to the antioxidant agents rather than unsaturated fats. Furthermore, antioxidant agents are present in extra virgin olive oil and not in olive oil. There is some evidence, based on studies using patients, to support such a supposition. In this paper, we present a case report on the possible health effects of changing from olive oil to extra virgin olive oil. The case report is intended to raise some relevant issues to stimulate a debate and more research in this area.

## INTRODUCTION

Current studies suggest that the type of fat rather than the quantity of fat consumed is directly related to levels of blood cholesterol[1]. The reported link between coronary heart disease (CHD) and cholesterol levels has led to a recommendation to increase consumption of oils high in unsaturated fats in the diet of the general population[2,3,4]. For example, expert reports from the WHO and the American Medical Association recommend increased consumption of fruit, vegetables, and unsaturated oils.

- http://www.who.int/hpr/NPH/docs/gs_chronic_disease.pdf
- http://www.who.int/mediacentre/releases/2003/pr20/en/
- http://www.ama-assn.org/ama/pub/printcat/11004.html

Furthermore, in the high risk population (those with high blood cholesterol levels), various medical regimes and interventions are made to control their cholesterol levels[5,6]. Generally, a Mediterranean type diet is recommended to control cholesterol levels and reduce the risk of CHD.

The relationship between a Mediterranean type diet and lower mortality ratio due to CHD has been attributed to monounsaturated fat[7] (oleic acid in olive oil). It is assumed that monounsaturated fatty acids may lower blood cholesterol levels and may increase high density lipoprotein (HDL)-cholesterol levels, which may be a link between olive oil consumption and the lower incidence of CHD[8]. It is not always clear if the resistance to oxidation from consuming the Mediterranean diet is due only to oleic acid and/or to other nontriglyceride components present in oleic acid–rich oils[8].

The minor constituents of virgin olive oil are nonglycerides such as hydrocarbons, monoglyceride esters, tocopherols, hydroxy and dihydroxy terpenic acids, sterols, polyphenols, and phospholipids[9]. The level of these compounds in olive oil is dependent on several agronomic factors including soil, degree of olive ripeness, and cultivar or olive variety[9]. Among the phenolic compounds present in olives, hydroxytyrosol is a diphenolic compound common in extra virgin olive oil[10]. Hydroxytyrosol has been shown to be highly protective against oxidative compounds that damage DNA *in vitro*[10]. *In vitro* incubation of LDL with phenolic compounds (Oleuropein or hydroxytyrosol) decreases vitamin E oxidation[10]. Also the phenolic compounds prevent the oxidation of linoleic and docosahexaenoic compounds in the LDL phospholipids. Phenols can also inhibit platelet aggregation[11].

Olive oil with high monounsaturated fatty acid (MUFA) content and sunflower oil with high polyunsaturated fatty acid (PUFA) content have been compared in the diet of type II diabetic people by a randomized 2-week crossover study. The results showed that cholesterol and LDL-cholesterol blood levels were significantly higher with sunflower than olive oil, suggesting that olive oil contributed to the lowering effect of cholesterol in the blood[12].

It is hypothesized that oleic acid in olive oil has a lowering effect on cholesterol in blood. Ruiz-Gutierrez and colleagues[13] examined the effect of olive oil and sunflower oil with the same proportion of oleic acid on normo–lipidemic and normo-tensive subjects. Their studies showed that sunflower oil with a similar amount of MUFA did not have the same effect as the olive oil, suggesting that there are other factors in olive oil that contribute to the lowering action of lipid profile.

Thus far, we can ascertain from the literature that there is a wide variation between similar oils in terms of their composition. The question is whether these differentials in oil components may affect human health. Furthermore, the general consensus on a balanced diet hardly distinguishes between oils over and above saturated/unsaturated fats. For example, see:

- http://www.who.int/hpr/NPH/docs/gs_chronic_disease.pdf
- http://www.who.int/mediacentre/releases/2003/pr20/en/
- http://www.ama-assn.org/ama/pub/printcat/11004.html

However, to our knowledge, there have not been any trials or experiments with the healthy population to investigate variations in blood cholesterol due to a change in the type of oil consumed. In order to stimulate debate and encourage more research in this area we would like to present the case of a healthy adult, who made a change to extra virgin olive oil from previously using ordinary olive oil.

## CASE REPORT

The case is a male individual in his mid-forties, generally physically active with stable dietary habits (see Appendix for a 1-week diet recall) and no history of major health problems or illnesses requiring a blood test for cholesterol levels. He had experimented with a number of different cooking oils including grapeseed oil, sunflower, walnut, and ordinary and extra virgin olive oil. Prior to the blood test at baseline, for a number of years the main oil consumed by the subject was ordinary olive oil. The knowledge by the subject about consumption of olive oil was what had been purported in the media about the Mediterranean diet and consumption of unsaturated oils. Therefore, the assumptions had been that other than taste and aroma, there was no difference in the health benefits between extra virgin olive oil and ordinary olive oil.

In February 2002, the results of blood tests that included cholesterol levels for the case became available (baseline measures) for the first time. By March 2002, the subject had made a 100% change to

**TABLE 1**
**Details of the Blood Cholesterol Tests at Two Time Points**

| Lipid profile | Base-line (mmol/l ) 19-02-02 | Follow-up (mmol/l) 30-07-03 | NHF REC* |
|---|---|---|---|
| HDL-C | 1.8 | 1.8 | > 1 |
| LDL-C | 3.8 | 3.4 | < 3.5 |
| Triglyceride | 0.7 | 0.5 | <2 |
| Total | 5.8 | 5.4 | 3 - 5 |

* New Zealand National Heart Foundation Recommended Ranges extra virgin olive oil. In July 2003, another blood test for cholesterol levels of the case became available (first follow-up measures). The results of the blood tests are shown in Table 1. A total blood cholesterol of 5.8 was recorded for the case at baseline. However, not knowing anything at all about the case's cholesterol levels past performance, not much could be said about these results other than that a total cholesterol of 5.8 was apparently above average. Given the general diet of the subject, the recorded cholesterol levels were rather disconcerting.

The results recorded on July 30, 2003 (Table 1) showed a decrease in total cholesterol of 0.4.

## DISCUSSION

As discussed earlier, any independent health effects of olive oil need to be studied in an experiment involving healthy individuals where such an effect could be isolated. This example presented precisely such a study as a "case". The advantage of this case is that, for all intents and purposes, we can ignore the placebo effect simply because the case was unaware of any differences between the oils. The question that arises is whether such an effect can be detected in the general population of healthy individuals.

It has been shown that polyphenolic compound level decreases as chemical extraction and purification of extra virgin olive oil proceeds[14]. Other benefits of extra virgin olive oil, possibly through enhanced nitric oxide levels stimulated by polyphenols, may be a reduced medication dosage requirement. For example, compared with those patients on a PUFA diet, those on a diet of extra virgin olive oil required less antihypertensive medication dosage[15]. However, the level of antioxidants, oleic acid, and other components in olive oil vary depending on the olive maturation and varieties, as well as the method of processing[16].

Experimental work suggests the phenolic compounds in olive oil may reduce oxidizability of LDL[17]. Other studies suggest that the daily ingestion of virgin olive oil could protect LDL from oxidation[18,19,20], and that its health benefit in terms of cardiovascular protection should not be ignored[21,22,23].

The link between cholesterol levels and CHD is currently being debated in the media, the popular press, Internet pages, and scientific journals. In theory, debate is healthy and a natural step in the dissemination of research results. It should lead to the identification of ways and methods to further our understanding of the process under investigation. However, often a division among the scientists about the value of the evidence is inevitable, when practice is seen to be strongly based on it.

While, with the current evidence, a link between type of oil and blood cholesterol may be established, the evidence linking blood cholesterol levels to CHD is not as clear cut[24]. The relationship between fat and CHD appears to be by association through the relationship between fat and cholesterol levels. Thus, medical interventions to control cholesterol levels in individuals may be influenced by the assumption that unsaturated fats lower blood cholesterol and therefore unsaturated fats lower the risk of CHD[5,6].

Some authors suggest no observed link[25], while others[26,27] report a link between cholesterol levels and CHD. Some suggest that cholesterol levels may not have a direct and independent effect on

CHD, but the link could well be the result of interaction effects[27]. Some other studies also link the lowered cholesterol levels to depression and suicide[28,29].

Originally, in this case, the change in diet was not part of any systematic study or experiment. Therefore, regular blood tests were not planned. As mentioned earlier, we have no knowledge of the past cholesterol levels of the subject. So it is difficult to make any comments about the dynamics of cholesterol levels, nor can we comment about the time taken for the change in the cholesterol levels to occur. Some questions that arise are:

- Would cholesterol levels still have gone down had the subject not changed to extra virgin olive oil? If so, by how much?
- Is it a coincidence that during the same period the cholesterol levels of the wife also decreased?
- Would the effect (if any) be mainly on the LDL cholesterol?
- How much of this change can be attributed to the change in oil?
- Can we expect a similar change in cholesterol across the healthy population?
- What are the dynamics of cholesterol in a healthy population (within and between individual variations), i.e., how does the cholesterol level vary over a period of time? How does it vary between individuals?
- What factors govern the dynamics of cholesterol?
- How much of the variation could be attributed to systematic effects such as a change in diet?
- How much is “safe” consumption of extra virgin olive oil?
- Should we expect the same or similar effects from the consumption of fresh olives (fruit) and/or other fruits and vegetables?

To address some of these questions, we have recently designed and written a research proposal. Outcomes of such research will have far reaching implications for the whole society. If there is a link between cholesterol levels and CHD, then a simple dietary change will have health and economic benefits for the health services and the population, it will have impact on the national income, farming and food production, manufacturing, and employment. On the other hand if the CHD-cholesterol link is not as important as first thought, then such a simple dietary change for controlling cholesterol levels is a cheap and effective method of empowering individuals to be in control of their health.

## BIOSKETCHES

**Shahnaz Shahtahmasebi** is currently a research methodology consultant and statistician at Christchurch Polytechnic Institute of Technology, Christchurch, New Zealand. His work experiences are mainly in the U.K. and cover a number of fields including public health, mental health, nursing, operational research, food, nutrition, and gerontology. His area of interests and expertise are related to longitudinal modeling of health related behavior. He has set up the Good Life Project in New Zealand, which attempts to investigate food holistically.

**Shahnaz Shahtahmasebi** is a food scientist currently lecturing on nutrition and dietetics in the School of Clinical Sciences, Charles Sturt University, Wagga Wagga, Australia. Her research interests relate to nutritional behavior and human health. She received her Ph.D. in food science from Leeds University, U.K (1982).

## APPENDIX – 1 WEEK DIET DIARY

### Prebaseline

***Sunday***. Breakfast — cereal with added raisin, banana, and full fat milk. Lunch — one large egg with tomatoes and garlic fried in ordinary olive oil with pitta bread. Dinner — grilled ground top side of beef and onion with rice, butter, and egg yoke. Supper — one apple, one pear, and an orange.

***Monday***. Breakfast — two pieces of toast with jam/honey. Lunch — homemade tuna sandwich, salad, an apple, and a piece of cake. Dinner — pasta with ground top side of beef in a tomato and vegetable (including peas, mushrooms, carrots) sauce. Supper — one apple, one pear, and an orange.

***Tuesday***. Breakfast — two pieces of toast with jam/honey. Lunch — homemade egg (boiled) sandwich, salad, an apple, and a piece of cake. Dinner — grilled chicken with vegetables, salad, and bread. Supper — one apple, one pear, and an orange.

***Wednesday***. Breakfast — two pieces of toast with jam/honey. Lunch — cheese sandwich, salad, an apple, and a piece of cake. Dinner — chicken in a curry sauce with rice and salad. Supper — one apple, one pear, and an orange.

***Thursday***. Breakfast — two pieces of toast with jam/honey. Lunch — homemade tuna sandwich, salad, an apple, and a piece of cake. Dinner — Mexican style chili with tortillas with ground top side of beef. Supper — one apple, one pear, and an orange.

***Friday***. Breakfast — two pieces of toast with jam/honey. Lunch — homemade cheese sandwich, salad, an apple, and a piece of cake. Dinner — sauté chicken with stir-fry vegetables with rice and salad. Supper — one apple, one pear, and an orange.

***Saturday***. Breakfast — one fried egg with toast (two pieces) with butter and jam/honey. No Lunch. Dinner — takeaway-chicken on a bed of salad in souvlaki bread. Supper — a piece of cake, one apple, and an orange.

### Postbaseline

There is very little difference between the pre- and postbaseline diet except that in the postbaseline dietary regime all sauces, marinades, and frying are prepared in extra virgin olive oil as opposed to ordinary olive oil as in the prebaseline diet. On average, one litre of oil would last a fortnight.

The variations from week to week are merely how the ingredients are cooked. There is no set pasta day but, on average, pasta is cooked once a week and it may be lasagne, spaghetti, macaroni, etc. Overall, the average diet before and after baseline has not changed considerably. The only change in the diet beyond the baseline is the cooking oil; the use of extra virgin olive oil in all cooking as opposed to ordinary olive oil.
